# Biodegradation of Sewage Wastewater Using Autochthonous Bacteria

**DOI:** 10.1100/2012/861903

**Published:** 2012-01-04

**Authors:** Purnima Dhall, Rita Kumar, Anil Kumar

**Affiliations:** ^1^Environmental Biotechnology Division, Institute of Genomics and Integrative Biology, Mall Road, Delhi 110007, India; ^2^Patent Division, National Institute of Immunology, Aruna Asaf Ali Marg, New Delhi 110067, India

## Abstract

The performance of isolated designed consortia comprising *Bacillus pumilus, Brevibacterium sp, and Pseudomonas aeruginosa* for the treatment of sewage wastewater in terms of reduction in COD (chemical oxygen demand), BOD (biochemical oxygen demand) MLSS (mixed liquor suspended solids), and TSS (total suspended solids) was studied. Different parameters were optimized (inoculum size, agitation, and temperature) to achieve effective results in less period of time. The results obtained indicated that consortium in the ratio of 1 : 2 (effluent : biomass) at 200 rpm, 35°C is capable of effectively reducing the pollutional load of the sewage wastewaters, in terms of COD, BOD, TSS, and MLSS within the desired discharge limits, that is, 32 mg/L, 8 mg/L, 162 mg/L, and 190 mg/L. The use of such specific consortia can overcome the inefficiencies of the conventional biological treatment facilities currently operational in sewage treatment plants.

## 1. Introduction

Until the last 200 years or so, the deterioration of watercourses due to organic pollution was not a serious problem because a relatively small human population lived in scattered communities and the wastes dumped into rivers could be coped with, by the natural self-purification properties. Water pollution became a severe problem with the industrialization of nations, coupled with the rapid acceleration in population growth. Industrialization led to urbanization, with people leaving the land to work in the new factories. Domestic wastes from the rapidly expanding towns and wastes from industrial processes were all poured untreated into the nearest rivers. Effluent waters, which should be removed from settlements and industrial enterprises, are known as sewage. Effluents are classified by their origin as domestic or public sewage, industrial effluents, and atmospheric (rain) run off. The sanitary requirements for the composition and properties of water bodies appreciably limit the discharge of sewage into water bodies. 

The term “sewage sludge” or “biosolids” represents the insoluble residue produced during wastewater treatment and subsequent sludge stabilization procedures, such as aerobic or anaerobic digestion [1]. The term sewage refers to the wastewater produced by a community which may originate from three different sources: (a) domestic wastewater, (b) industrial wastewater, and (c) rain water.

Depending on the degree of pollution and the sanitary requirements, all effluents can be either discharged straight into a stream or only after the appropriate treatment (mechanical, chemical, or biological). The sewage should be specially treated before disposal. The method of sewage treatment fall into two groups, mainly, destructive and regenerative methods. Vymazal and Kr*ӧ*pfelová [2] studied the effect of three-stage experimental constructed wetlands for the treatment of sewage. Reported that 94.5% of BOD and 84.4% of the COD were removed. Microbial fuel cell with ultra sound pretreatment was assessed by Jiang et al. [3], and it was observed that from raw sewage TCOD removal rate was 11.3% to 19.2% and in case of pretreated sludge it was 25% to 57%. An integrated UASB-sludge digester system was observed in temperate climates [4], and it was seen that, with decrease in temperature, the COD removal decreased from 78% at 28°C to 42% at 10°C. On the other hand, Sabry 2008 [5] studied the application of UASB inoculated with flocculent and granular sludge for treating sewage. After 4 h of retention time, 3-4% of the COD was removed. Waleed et al. [6] use the cloth media filter membrane bioreactor for treating municipal wastewater, and it was observed that 93–95% of COD, 99% of TSS, and turbidity 89–94% were removed with a retention time of 26.3 days. Garcia et al. [7] studied effects of bed materials on the performance of an anaerobic sequencing batch biofilm reactor that was used for treating the domestic sewage; results were analyzed, and it was revealed that the removal efficiencies for CODtot, CODsus, BOD_5_, and TSS were 56%, 87%, 59%, and 81% for R1 and 58%, 90%, 60%, and 82% for R2, respectively. Domestic sewage treatment in a pilot-scale anaerobic sequencing batch biofilm reactor was observed by Sarti et al. [8]. Data obtained from 125 days of treatment was analyzed, and it was seen that, under stable operating conditions after the startup period, the mean values of COD removal efficiency achieved was 66%. The comparative performance of three pilot-scale anaerobic sequencing batch reactors treating domestic sewage was evaluated, and it was seen that ASBR1 and ASBR3 reactors operated under mixed liquor recirculation showed nonsatisfactory results, attaining mean values of COD and TSS removals efficiencies of 40% and 65%, respectively. The ASBR2 operated under mechanical mixing showed better results with average removal efficiencies of 60% and 80% for COD and TSS, respectively. Rosal et al. [9] oxidized the effluent from secondary clarifier of an urban sewage treatment plant by using ozone and hydrogen peroxide. The removal efficiency of total organic carbon was no higher than 35%. Elmitwalli et al. [10] use a column packed with clean sheets of reticulated polyurethane foam and fed with either raw sewage, synthetic sewage, or skimmed milk for a short time to evaluate the mechanism of physical entrapment and filtration of particles. The results revealed that clean media were effective in the removal of suspended chemical oxygen demand (COD) (>75%). Wang [11] found that the removal of colloidal particles was the rate-limiting step in a two-step UASB + EGSB system treating domestic sewage at a HRT of 3 + 2 h under low-temperature conditions. He found average removal efficiencies for colloidal COD (CODcol) of 40% and 49% at temperatures of 17°C and 12°C, respectively, Yoda et al. [12] reported that the colloidal particles in the influent were very difficult to remove and represented up to 60 ± 70% COD of the effluent of an anaerobic fluidized bed (AFB) reactor. Shivarajum [13] describes the photocatalytic degradation of organic pollutants present in the municipal sewage water by using hydrothermally prepared supported photocatalyst. The results obtained represented that the organic pollutants removal efficiency reached upto 97% for 8 h irradiation time.

Abdulaziz et al. [14] evaluate the use of membrane bioreactor in combination with activated sludge for the reduction of BOD and COD from sewage wastewater. Results showed reduction in 98.5% of COD and 96% of BOD. Similar findings were reported by many researchers by using sedimentation, aeration, activated sludge, sand filters and activated carbon [15], and low-cost carbonaceous materials [16] for the reduction of COD and BOD from wastewater. The reduction of COD ranging from 92.1 to 99.05% and BOD from 98.9 to 99% was reported at different places. The major disadvantage of using activated sludge for the treatment is generation of large amount of sludge which itself is a major problem for disposal.

The selection of a particular method of treating sewage depends on the composition and properties of the sewage and also on the character and the capacity of the water body. Biological treatment is necessary if organic matter is to be removed from water. Nonetheless, biological treatment offers an economical alternative to physical and chemical treatment methods. It is the most widely used method for removal as well as partial or complete stabilization of biologically degradable substances present in wastewaters. The mechanism underlying biological treatment is the decomposition of finely dispersed matter, colloidal and dissolved substances by metabolism of aerobic microorganisms. The susceptibility of organic substances contained in sewage, to biochemical oxidation coupled with the presence of specific biooxidation agents, that is, microorganisms, is a prerequisite for efficient biodegradation. Specific microorganisms may be required to biodegrade the organic contaminants present in sewage. Employment of single microorganisms may not suffice the purpose. A mixture of microorganisms may have a cumulative effect on increasing the biomass activity, growth efficiency, and enzyme production. In addition, mixed cultures serve to overcome feedback regulation and catabolic repression, as the products of one microorganism act as substrate for the other.

Effective sewage treatment prevents a variety of ailments that can be spread by exposure to pathogens that can be present in untreated sewages and thus helps prevent disease. Discharges of untreated sewage can contaminate ground waters and surface waters used for drinking, recreation, and fish and shellfish fisheries.

Untreated sewage from failed conventional septic systems or sewage discharged directly into the environment can percolate into groundwater, contaminating drinking-water wells with pathogens. The discharge of untreated sewage to streams can spread disease through direct contact, making such streams unfit for forms of recreation that involve skin contact with the water such as swimming and boating. Disease can also spread by indirect (secondary) contact such as through contact with rodents or insects that received primary exposure and in turn harbor the pathogens. Discharged, untreated sewage also can damage the receiving streams' ability to support healthy, living communities of aquatic organisms and can contaminate fisheries. 

In the present work, a biotechnological approach has been utilized to treat the sewage wastewater using specific bacteria having biodegradative potential for sewage wastewater. Biodegradation of sewage wastewater was studied in terms of reduction of COD, BOD, MLSS, and TSS.

## 2. Material and Methods

### 2.1. Collection of Soil Samples

Soil samples were collected in sterile plastic containers from niche areas near the Vasanthkunj sewage treatment plant, New Delhi, for the isolation of bacterial isolates.

### 2.2. Isolation of Bacteria

Isolation of bacteria was done by using serial dilution method. For bacterial isolation, soil extract and enrichment media were prepared. The soil extract was prepared by dissolving 300 g of soil sample in 1200 mL distilled water to make dilution. The solution was centrifuged at 8000 rpm for 15 min to remove the coarse material, after autoclaving at 15 psi for 15 min. The enrichment media was prepared by transferring 5 g of soil to a conical flask (500 mL) containing 125 mL soil extract and 125 mL of nutrient broth followed by the addition of 36 *μ*L of antifungal. The flask containing enrichment media was incubated at 37°C in a rotary shaking incubator at 120 rpm for 2 days. The well-vortexed enrichment media was serially diluted with autoclaved saline water (0.85% NaCl) to 10^−1^, 10^−2^, 10^−3^, 10^−4^, 10^−5^, and 10^−6^ dilutions. Diluted sample spread on different designed media plates (Table 1). Plates were incubated at 37°C for 16 h.

### 2.3. Formulation of Consortia

A consortium is a group of specific bacterial isolates which possesses the capability to degrade the components present in the wastewater. For preparation of one consortium, we have used three to four different isolates. Different consortia were formulated randomly primarily on the basis of their morphology, color, size, shape, and so forth.

### 2.4. Screening of Different Consortium for Bioremediation

Different cultures were inoculated in 25 mL of NB and incubated overnight at 32–37°C and 180–200 rpm. These mother cultures were checked by streaking on nutrient agar plates which were then incubated at 32–37°C. These mother cultures were used for subculturing. 100 *μ*L of culture was inoculated into 100 mL of NB and incubated at 32–37°C under shaking conditions for a period of 16–18 h. The culture was harvested by centrifugation at 4°C and 7000 rpm followed by washing twice with sodium phosphate buffer (pH 6.8–7.0). The supernatant was discarded and pellets were stored for the further experiments. At the time of experiment, different pellets were resuspended according to the 20 consortia designed and inoculated in the sample and sample flasks were kept in shaking incubator at 180–200 rpm and 32–37°C for 32–36 h. After incubation, COD/BOD was estimated according to the procedure mentioned in standard methods (APHA) [17].

### 2.5. Bioremediation by Using Selected Consortium

Cultures were prepared according to the procedure mentioned above. Pellets were resuspended at the time of experiment, and consortium number 13 was inoculated in the sample, and sample flasks were kept in shaking incubator at 180–200 rpm and 32–37°C for 32–36 h. After incubation, the COD, BOD, MLSS, and TSS were estimated according to the procedure mentioned in standard methods (APHA) [17].

### 2.6. Identification of Selected Bacterial Isolates

The selected organisms of the consortium were identified by 16s rRNA studies at IMTECH, Chandigarh. Morphological, physiological and biochemical tests were also carried out [18] by the identification service of MTCC (Chandigarh, India).

Although 16S rRNA gene is found to be conserved on evolutionary scale, it is still diverse enough for identifying and classifying the eubacteria [19]. For 16S rRNA sequencing, the bacterial culture was inoculated in Luria Bertani broth (Himedia). Overnight-grown bacterial culture was used for total DNA isolation using Genomic DNA Extraction kit. (Real Biotech Corporation). 16 rRNA gene was amplified using universal primers. The PCR reaction mixture contained assay buffer 5 *μ*L, forward primer 1 *μ*L, reverse primer 1 *μ*L, dNTP 1 *μ*L, template 2 *μ*L, and tag polymerase 1 *μ*L, and final total volume was makeup 50 *μ*L with milli Q. Polymerase chain reaction was performed in a thermocycler (BIORAD) under the following conditions: denaturation at 94°C for 1 min, followed by annealing at 55°C for 1 min, and extension at 72°C for 2 min, for 35 repeated cycles. Approximately 1500 bp region of the gene was amplified, and the amplification product was gel purified using QIA gel extraction kit and sequenced. The sequence data was analyzed by BLAST and identified based on closest similarity with the reported sequenced data.

### 2.7. Optimization of Parameters

After the whole experiment, the different parameters like bacterial biomass, shaking speed, temperature, and so forth need optimization. Various parameters (temperature and agitation) were standardized in order to get efficient treatment in less duration.

### 2.8. Achieving Short Retention Time by Cyclic Treatment of Sewage Wastewater

**Figure 1:**
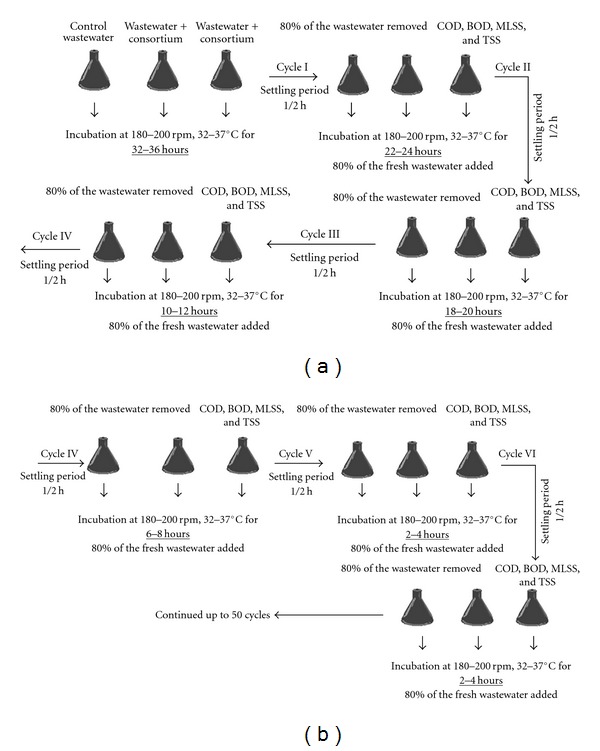
Continuous process for reducing time of degradation there by reducing COD, BOD, MLSS, and TSS.

In order to reduce the time of degradation, the consortium was acclimatized as follows. Culture was grown, and pellet was resuspended as mentioned above and inoculated in sample flask. The flask was incubated in shaker at 180–200 rpm, 32–37°C for 32–36 h. After 36 h, the COD was estimated, and, after 30 min of settling, 80% of the sample was removed and replaced with fresh sample. Flask was again kept for shaking at 200 rpm for 32–36 h, and the COD was estimated after every 4 h. The same process was repeated, and thus the incubation time was gradually reduced to 20, 12, 8, and finally to 4 h (Figure 1).

## 3. Result and Discussion

### 3.1. Isolation and Characteristics of Bacterial Population

36 bacterial isolates were purified from all the above-mentioned isolation procedure. It was hypothesized that bacteria isolated from their natural habitat have capability of surviving in harsh conditions by developing some catabolic enzymes systems, specific for particular components present in the natural habitat. The isolated colonies were diverse in their morphologies, ranging from small pin-pointed to large sized, smooth margined to wrinkled periphery, shining to dry, and so on. Supplementary Table  1 available online at doi: 10.1100/2012/861903 shows the morphological characteristics of those isolated bacterial population obtained during the process of isolation.

### 3.2. Formulated Consortia

A consortium is a group of specific bacterial isolates which possesses the capability to degrade the components present in the wastewater. For preparation of one consortium, we have used three to four different isolates. 15 different consortia were prepared and checked for their biodegradative potential for sewage wastewater. Different consortia were formulated randomly primarily on the basis of their morphology, color, size, shape, and so forth. The formulated consortia are depicted in Supplementary Table  2.

### 3.3. Bioremediation Studies

**Figure 2:**
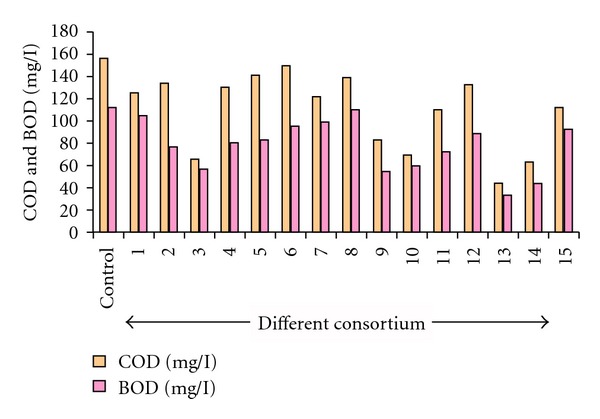
Comparison of BOD and COD for 15 consortia.

**Figure 3:**
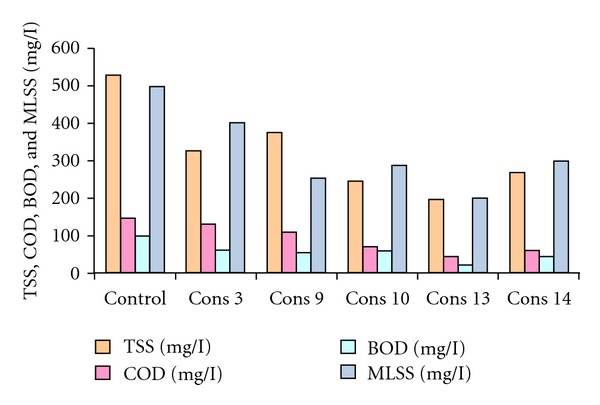
Comparison of TSS, COD, BOD, and MLSS of five selected consortia.

Formulated consortia were tested for their COD/BOD reduction potential using sewage wastewater sample. The results of these experiments showed that, after 36 h of incubation, consortia 3 shows percentage degradation of 50%, consortia 9 shows degradation up to 51%, and similarly the rest of the selected consortia 10, 13, and 14 show degradation of 46%, 70%, and 60.7% in comparison to other consortia which show degradation in the range of 1.79 to 35.7%. COD/BOD reduction achieved in different flask was mentioned in Figure 2. Five consortia (3, 9, 10, 13, and 14) were selected, and the above-mentioned experiment was repeated after 36 h COD/BOD/MLSS/TSS was estimated, and the results reveal that the consortium 13 gives the best results. It shows percentage degradation of 78% in comparison of other consortia (Figure 3). The reduction achieved with consortium 13 after 36 h of incubation in case of COD, BOD, MLSS, and TSS was 148–44 mg/L, 100–22 mg/L, 500–199 mg/L, and 525–198 mg/L. The results of this cycle with rest of the consortia (3, 9, 10, and 14) show the reduction in COD up to 131 mg/L, 110 mg/L, 70 mg/L, and 62 mg/L, BOD up to 61 mg/L, 52 mg/L, 60 mg/L, and 45 mg/L, MLSS up to 400 mg/L, 251 mg/L, 287 mg/L, and 300 mg/L and TSS up to 325 mg/L, 374 mg/L, 244 mg/L, and 269 mg/L, respectively. Consortium 13 was selected for further studies.

### 3.4. Identification of Cultures

Strains were identified on the basis of physiological, morphological, biochemical, and 16rRNA techniques performed at MTCC (Chandigarh). Strains of the selected consortium 13 were identified as *Bacillus pumilus, Brevibacterium sp, *and* Pseudomonas aeruginosa.*

Morphological characteristics (margin, elevation, surface, opacity, gram's reaction, cell shape, endospore, position, shape, motility, and fluorescence) of the identified strains is depicted in Table 2.

Physiological tests and various biochemical tests were also performed, and the results showed that *Bacillus pumilus, *MTCC (5305) is aerobic in nature, gram positive, motile, shows its growth from 25 to 45°C, capable to starch hydrolysis, and catalase positive. *Brevibacterium sp. *(MTCC 5306) is aerobic in nature, gram positive, motile, shows its growth from 25 to 45°C catalase and oxidase positive. *Pseudomonas aeruginosa *(MTCC 5307) is aerobic in nature, gram negative, motile, shows its growth from 25 to 42°C, this bacterium capable to utilize the citrate and hydrolyze casein, catalase, and oxidase positive (Tables 3 and 4).

### 3.5. Optimization of Parameters by Using Consortium 13

**Figure 4:**
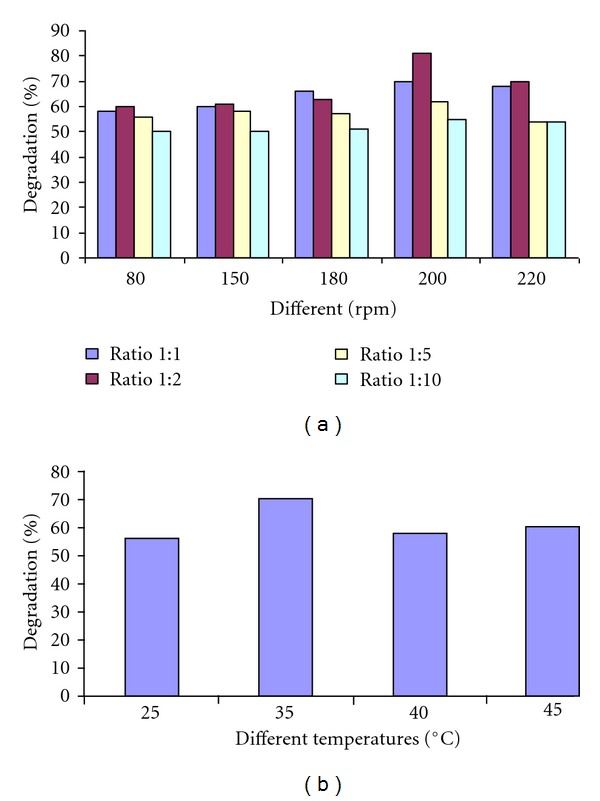
(a) Comparison of percentage degradation of sewage sample after 8 h at different shaking speed and by using different inoculum size of consortium 13. (b) Comparison of percentage degradation of sewage sample after 4 h at different incubation temperature.

Effect of inoculum size and Agitation: the effect of biomass on the COD reduction ability of consortium was studied. Different effluent: biomass ratios of 1 : 1, 1 : 2, 1 : 5, and 1 : 10 were tried. Agitation was tested simultaneously; flasks were incubated at different rpm (80 rpm, 150 rpm, 180 rpm, 200 rpm, and 220 rpm). Results suggested that consortium 13 produced the best results when used in the 1 : 2 effluent: biomass ratio and incubated at 200 rpm. The reduction in COD was observed up to 79% after 36 h of incubation period (Figure 4(a)).

**Temperature**

Different temperatures were also studied for better COD reduction. The results reveal that better COD reduction could be achieved in the flask incubated at 35°C as compared to the other flasks incubated at 25°C, 40°C, and 45°C (Figure 4(b)).

### 3.6. Cyclic Treatment of Sewage Wastewater

The selected consortium comprises 3 bacterial isolates found to be capable of reducing COD, BOD, and TSS of sewage wastewater. In order to reduce the time of degradation, the consortium was acclimatized.  Table 5 shows the reduction in COD, BOD, and TSS by using selected consortium 13 while reducing time of incubation from 36 h to 4 h. The reduction achieved after 36 h in case of COD, BOD, MLSS, and TSS was from 196–48 mg/L, 78–15 mg/L, 425–170 mg/L, and 546–200 mg/L. After 30 min of settling, 80% of wastewater was removed and fresh 80% was added, and the sample was incubated for 24 h. The results of this cycle show the reduction in COD from 172 to 40 mg/L, BOD from 68 to 10 mg/L, MLSS 600 to 225 mg/L, and TSS from 624 to 198 mg/L. In the same manner, settling was done for 30 min and incubation time was further reduced to 20 hr. Estimation revealed the reduction 152–32 mg/L, 62.8–11 mg/L, 525–191 mg/L, and 569–190 mg/L in COD, BOD, MLSS, and TSS, respectively. This reduction in incubation time was carried on further from 12 h to 8 and then finally to 4 h. The final results show that the percentage degradation was 79% (155–32 mg/L), 85.5% (55–8 mg/L), 67.6% (500–162 mg/L), and 67.1% (578–190 mg/L) for COD, BOD, MLSS, and TSS, respectively, after 4 h of incubation. 

## 4. Conclusion

The selected formulated bacterial consortium comprising of the isolated bacterial strains acts in a synergistic way and is capable of degrading the easily assimilable organic compounds present in sewage wastewater. This consortium is capable of effectively reducing the pollutional load of the sewage wastewaters, in terms of COD, BOD, MLSS, and TSS within the desired discharge limits, that is, 32 mg/L, 8 mg/L, 162 mg/L, and 190 mg/L. The use of such specific consortia can overcome the inefficiencies of the conventional biological treatment facilities currently operational in sewage treatment plants.

## Supplementary Material

The supplementary material describes the isolation of autochthonous bacteria using different media plates (100% Soil extract media, 50% soil extract media, soil extract & nutrient broth media, nutrient agar media) (Table 1). Table 2 represents the formulation of different consortia. A consortium is a group of specific bacterial isolates which possesses the capability to degrade the components present in the wastewater. Different consortia were formulated randomly primarily on the basis of their morphology, color, size, shape and so forth. The file containing edited supplementary material is being uploaded along with these comments.

## Figures and Tables

**Table 1:** The composition of different media designed.

S. no.	Media composition
1	500 mL soil extract + 2% Agar
2	250 mL soil extract + 250 mL water + 2% Agar
3	250 mL soil extract + 250 mL nutrient broth + 2% agar
4	500 mL nutrient agar

**Table 2:** Morphological characteristics of the identified strains.

Test	*Bacillus pumilus*	*Brevibacterium sp.*	*Pseudomonas aeruginosa*
Colony morphology			

Margin	Irregular	Irregular	Irregular
Elevation	Convex	Convex	Flat
Surface	Dull	Glistening	Dull
Opacity	Opaque	Opaque	Translucent
Gram's reaction	+ve	+ve	−ve
Cell shape	Rods	Cocci/rods	Short rods
Endospore	+	−	−
Shape	Oval	−	−
Motility	+	+	+
Fluorescence (UV)	−	−	−

**Table 3:** Study of physiological features (pH, temperature, and NaCl concentration) of identified strains. (−) represents no growth and (+) growth.

Tests	*Bacillus pumilus*	*Brevibacterium sp.*	*Pseudomonas aeruginosa*
*Growth at* pH 4.0	−	−	−
pH 5.0	−	−	**+**
pH 6.8	**+**	**+**	**+**
pH 8.0	**+**	**+**	**+**
pH 9.0	**+**	−	**+**
pH 11.0	−	−	−

*Growth at* 4°C	−	−	−
10°C	−	−	−
25°C	**+**	**+**	**+**
30°C	**+**	**+**	**+**
37°C	**+**	**+**	**+**
42°C	**+**	**+**	**+**
45°C	**+**	**+**	**+**
55°C	−	−	−
65°C	−	−	−

*Growth on NaCl* (*%*)			
2.0	**+**	**+**	**+**
4.0	**+**	**+**	**+**
7.0	**+**	**+**	-
8.0	**+**	-	-
10.0	**+**	-	-

**Table 4:** Biochemical test of identified strains.

Tests	*Bacillus pumilus*	*Brevibacterium sp.*	*Pseudomonas aeruginosa*
Growth on MacConkey agar	−	−	+
Indole test	−	−	−
Methyl red test	−	−	−
Voges Proskauer test	−	−	−
Citrate utilization	−	−	**+**
Casein hydrolysis	−	−	**+**
Gelatin hydrolysis	−	−	−
Starch hydrolysis	**+**	−	−
Urea hydrolysis	−	−	−
Nitrate reduction	−	−	−
H_2_S production	−	−	−
Catalase test	**+**	**+**	**+**
Oxidase test	−	**+**	+

*Acid production from carbohydrates*			
Salicin	−	−	−
Arabinose	−	−	**+**
Galactose	−	−	**+**
Dextrose	**+**	−	**+**
Meso-Inositol	−	−	−
Raffinose	−	−	−
Rhamnose	**+**	−	−
Fructose	**+**	−	**+**
Mannitol	−	−	**+**
Sucrose	**+**	−	−
Xylose	−	−	**+**

**Table 5:** Showing the reduction in time thereby reducing the COD mg/L, BOD mg/L, TSS mg/L, and MLSS mg/L by using consortium 13.

Time		COD mg/L	BOD mg/L	TSS mg/L	MLSS mg/L
36 h	Control	196	78	546	425
	Consortium	48	15	200	170
	% degradation	**75.5**	**80.8**	**63.4**	**60**

24 h	Control	172	68	624	600
	Consortium	40	10	198	225
	% degradation	**76.7**	**85.3**	**68.3**	**62.5**

20 h	Control	152	62.8	569	525
	Consortium	32	11	185	191
	% degradation	**78.9**	**82.5**	**67.5**	**63.6**

12 h	Control	150	48	545	700
	Consortium	35	9	181	250
	% degradation	**76.7**	**81.3**	**66.8**	**64.3**

8 h	Control	168	59.6	600	656
	Consortium	35	9.6	212	230
	% degradation	**79.2**	**83.9**	**64.7**	**64.9**

4 h	Control	155	55	578	500
	Consortium	32	8	190	162
	% degradation	**79.4**	**85.5**	**67.1**	**67.6**
