# Supplementary material for: Biodegradation of Sewage Wastewater Using Autochthonous Bacteria
Source: ScientificWorldJournal. 2012 Jan 4;2012:861903. doi: 10.1100/2012/861903 (PMC3260589; doi:10.1100/2012/861903)
Supplement: Supplementary file 1 — The supplementary material describes the isolation of autochthonous bacteria using different media plates (100% Soil extract media, 50% soil extract media, soil extract & nutrient broth media, nutrient agar media) (Table 1). Table 2 represents the formulation of different consortia. A consortium is a group of specific bacterial isolates which possesses the capability to degrade the components present in the wastewater. Different consortia were formulated randomly primarily on the basis of their morphology, color, size, shape and so forth. The file containing edited supplementary material is being uploaded along with these comments. [file 861903.f1.pdf]

Supplementary Table ١ Morphological characteristics of isolated bacteria

S.N.	**Dilutions**	**Configuration**	**Margin**	**Elevation**	**Color**	**Size mm**
**Isolated bacteria from ١٠٠٪ Soil extract media**						
١	$١٠^{-٥}$	Round	Smooth	Convex	Bluish Cream	٣mm
٢	$١٠^{-٥}$	Round	Smooth	raised	White	pinpoint
٣	$١٠^{-٥}$	Round	Smooth	Drop Like	Yellow	٢mm
٤	$١٠^{-٥}$	Round	Smooth	Convex	Orange	٢mm
٥	$١٠^{-٥}$	Round	Smooth	Flat	Yellowish green	٤mm
٦	$١٠^{-٦}$	Punchiform	Smooth	Convex	bluish	pinpoint
٧	$١٠^{-٦}$	Round	Smooth	Raised	Orangish	١mm
٨	$١٠^{-٦}$	Round	Smooth	Convex	Yellowish green	٢mm
٩	$١٠^{-٦}$	Round	Smooth	Convex	Milky white	٢mm
١٠	$١٠^{-٨}$	Punchi form	Smooth	Convex	Bluish	Pinpoint
١١	$١٠^{-٨}$	Round	Smooth	Convex	Yellowish	٢mm
١٢	$١٠^{-٨}$	Irregular	Lobate	Umbonate	yellowish	٢-٣mm
**Isolated bacteria from ٥٠٪ Soil extract media**						
١	$١٠^{-٥}$	Round	Smooth	Raised	Creamy	١mm
٢	$١٠^{-٦}$	Round	Smooth	Convex	Whitish cream	Pinpoint
٣	$١٠^{-٦}$	Round	Smooth	Pulvinate	Orange	٢mm
٤	$١٠^{-٦}$	Round	Smooth	Raised	Orange	١mm
٥	$١٠^{-٦}$	Round	Smooth	Convex	Brown	٢mm
٦	$١٠^{-٧}$	Round	Smooth	Drop like	Yellowish	١mm
٧	$١٠^{-٧}$	Spindle	Smooth	Convex	Yellow	٢mm
٨	$١٠^{-٧}$	Round	Smooth	Drop like	Yellow glossy	١mm

٩	١٠-٩	Round	Smooth	Convex	White	١mm
**Isolated bacteria from Soil Extract & NB media**						
١	١٠-٥	Round	Smooth	Raised	Orange	١mm
٢	١٠-٥	Punchi form	Smooth	Convex	Pure white	Pinpoint
٣	١٠-٥	Round	Smooth	Drop like	Yellowish green	٢-٣mm
٤	١٠-٦	Round	Smooth	Pulvinate	Creamy white	١mm
٥	١٠-٦	Round	Smooth	Convex	Glossy orange	١-٢mm
٦	١٠-٧	Punchi form	Smooth	Convex	Yellow	Pinpoint
٧	١٠-٧	Round	Smooth	Convex	Milky white	٤-٥mm
٨	١٠-٨	Round	Smooth	Raised	Orangish	١-٢mm
٩	١٠-٨	Spindle	Smooth	Pulvinate	Thick white	٢mm
١٠	١٠-٨	Round	Smooth	Raised	Yellow	٢mm
١١	١٠-٩	Round	Smooth	Convex	Yellow	١mm
١٢	١٠-٩	Round	Smooth	Raised	Creamy white	٢mm
١٣	١٠-٩	Round	Smooth	Flat	Creamy white	٢mm
**Isolated bacteria from Nutrient Agar media**						
١	١٠-٦	Round	Smooth	Raised	White	٢mm
٢	١٠-٩	Round	Smooth	Convex	Thick white	٢mm

Supplementary Table ٢: Formulation of different consortia from isolated bacteria.

**Consortium**	**Bacterial strains**			
Consortia ١	SE ٢	SENB ٥	٥٠SE ٤	٥٠SE ٢
Consortia ٢	SE ٧	SE ٦	SENB ٧	٥٠SE ٥
Consortia ٣	SENB ١	SENB ٣	SENB ١١	SENB ١٢
Consortia ٤	SE ١	SE ٨	SENB ٩	٥٠SE ٥
Consortia ٥	SENB ٦	٥٠SE ١	٥٠SE ٧	NA ٢
Consortia ٦	SE ٧	SE ٩	٥٠SE ٤	٥٠SE ٥
Consortia ٧	SE ٣	SENB ١١	٥٠SE ٣	٥٠SE ١
Consortia ٨	SE ٥	SE ٤	SENB ١٢	٥٠SE ٥
Consortia ٩	SENB ١١	SENB ٩	SENB ١٠	٥٠SE ٦
Consortia ١٠	SE ١	SE ٤	SENB ١٠	٥٠SE ٥
Consortia ١١	٥٠SE ١	٥٠SE ٤	٥٠SE ٥	NA ١
Consortia ١٢	SE ٨	SE ٢	SENB ٤	SENB ١٢
Consortia ١٣	SE٦	٥٠SE٣	SENB١٢	SE١
Consortia ١٤	SE ٩	SENB ٧	SENB ١١	٥٠SE ٩
Consortia ١٥	SE ٦	SE ٩	SENB ١٠	٥٠SE ٦
